# Revealing noncollinear magnetic ordering at the atomic scale via XMCD

**DOI:** 10.1038/s41598-021-82518-4

**Published:** 2021-02-04

**Authors:** Fridtjof Kielgast, Ivan Baev, Torben Beeck, Federico Pressacco, Michael Martins

**Affiliations:** 1 Universität Hamburg, Institut für Experimentalphysik, Luruper Chaussee 149, 22761 Hamburg, Germany; 2 Deutsches Elektronensynchrotron (DESY) Photon Science, Notkestraße 85, 22607 Hamburg, Germany

**Keywords:** Magnetic properties and materials, X-rays

## Abstract

Mass-selected V and Fe monomers, as well as the heterodimer ${\text{Fe}}_1{\text{V}}_1$, were deposited on a Cu(001) surface. Their electronic and magnetic properties were investigated via X-ray absorption (XAS) and X-ray magnetic circular dichroism (XMCD) spectroscopy. Anisotropies in the magnetic moments of the deposited species could be examined by means of angle resolving XMCD, i.e. changing the X-ray angle of incidence. A weak adatom-substrate-coupling was found for both elements and, using group theoretical arguments, the ground state symmetries of the adatoms were determined. For the dimer, a switching from antiparallel to parallel orientation of the respective magnetic moments was observed. We show that this is due to the existence of a noncollinear spin-flop phase in the deposited dimers, which could be observed for the first time in such a small system. Making use of the two magnetic sublattices model, we were able to find the relative orientations for the dimer magnetic moments for different incidence angles.

## Introduction

Small magnetic adsorbate systems have been a focus of interest in recent years, as they are promising candidates for high-density data storage devices^1,2^. Beyond possible applications, the investigation of electronic and magnetic properties of adsorbates provides a way to study coupling phenomena on a fundamental level. It has been shown that the geometric constraints, imposed by a substrate, as well as the hybridization of the substrate with the adsorbate electronic states are often responsible for the appearance of large magnetic moments and anisotropies in magnetic adatoms^3^. Especially in 3*d* transition metals, a large number of interactions are present at comparable energy scales which leads to a complex behaviour and thus hinders theoretical predictions^4^. It is this complex interplay that is also at the basis of noncollinear magnetic systems.

A prominent example of such complex behaviour are Fe$_{1-x}$V$_{x}$ bulk alloys, which are found to exist in a complex tetragonal structure called the $\sigma$-phase in a V content range from x$\approx$30 at% to 60 at%^5^. It has been shown to behave as a re-entrant magnetic system which, upon lowering of the temperature, undergoes a transition from a paramagnetic to a ferromagnetic state and finally becomes a spin glass^6^. This calls for a closer look into the fundamental coupling between the two constituents. As a prototype model system, we prepared adsorbate systems with small amounts of the respective adatoms and the Fe$_1$V$_1$ dimer on a Cu(001) surface. The choice of Cu(001) is motivated by its *sp*-like density of states at the Fermi edge and the low-lying *d*-bands (see e.g.^7^), which provide the possibility for a weak substrate-adsorbate hybridization. Results found for Fe adatoms on Cu(111), where multiplet features have been observed, reinforce this^8^.

The element specificity of X-ray transitions makes XAS a well-suited technique to investigate the electronic structure of compound systems like the present one. Additionally, using circular polarized light makes the absorption signal sensitive to the magnetic moment of the probed element by X-ray magnetic circular dichroism (XMCD)^9,10^. We have chosen to investigate the $L_{2,3}$ edges, i.e. the 2$p_j\rightarrow 3d$ absorption (with $j=\frac{1}{2},\frac{3}{2}$) of V and Fe since their electronic and magnetic properties of are mainly determined by their 3*d* levels.

## Results and discussion

### Fe and V adatoms

**Figure 1:**
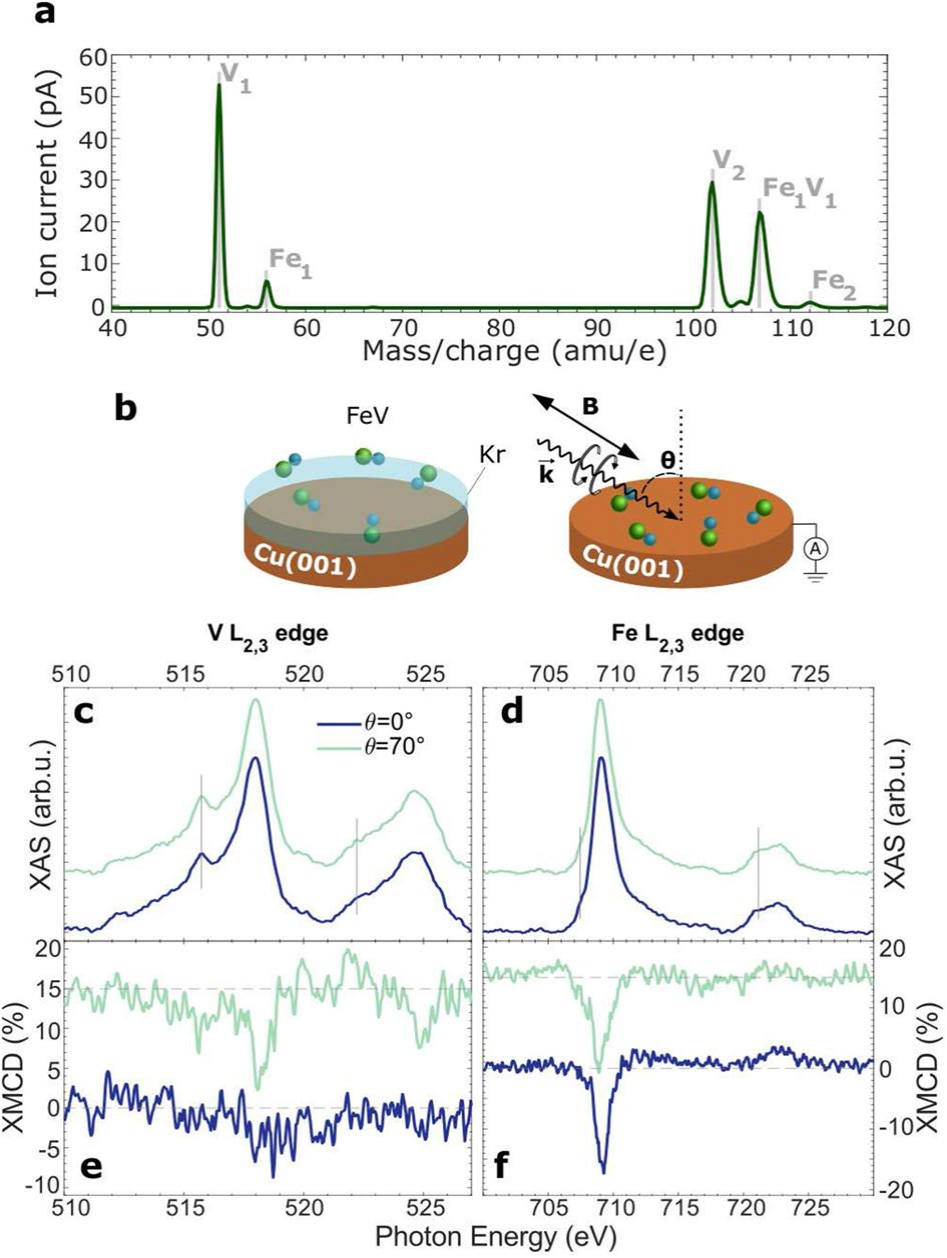
(**a**) Mass-to-charge spectrum of cluster ions up to n=2, produced from the FeV target. (**b**) Left: sketch of the soft landing procedure. Right: schematic view of the measurement geometry after the Kr buffer is removed by flash heating to 120 K. The angle $\theta$ between the surface normal and the incoming photon beam is varied between 0 $^{\circ }$ and 70 $^{\circ }$. The magnetic field strength *B* was alternated between $-7$ and $+7$ T. Background corrected white line spectra taken at $\theta$=0$^\circ$ and $\theta$=70$^\circ$ for V$_1$/Cu(001) (**c**) and Fe$_1$/Cu(001) (**d**). (**e**) and (**f**) are the XMCD spectra corresponding to (**c**) and (**d**). All spectra are normalized to the $L_3$ peak value of the corresponding white line spectrum and vertically offset for better visibility.

First, we will discuss the electronic properties of the Fe and V adatoms on Cu(001). The background corrected absorption spectra across their respective $L_{2,3}$-edges, as well as the resulting dichroism spectra are shown in Fig. 1c–f. In order to compare the spectral changes, these spectra have been normalized to their respective $L_3$ peak values. For both adatoms, the spectra show features at the lower energy flanks of the respective $L_{2,3}$-peaks ($\sim$515.7 eV and 522.2 eV for V$_1$ and $\sim$707.4 eV and 721.1 eV for Fe$_1$). These multiplet features are the result of the interaction of the 2*p* core hole and the 3*d* electrons and are typically found in atomic and atomic-like species^11–13^. Comparison of Fig. 1c–d with XAS of free Fe and V atoms (cf.^11^) shows very good agreement. The broadening and general energetic shift of the present spectra are due to the reduction of the Slater integrals, which is to be expected for systems showing some degree of hybridization^14^. The visibility of multiplet features and the resemblance of the spectra to those of free atoms implies a low degree of hybridization of the adatom *d*-states with the Cu(001) surface and ensures that the investigated magnetic properties are intrinsic to the adsorbates.

**Table 1:** Branching ratios of the adsorbates for $\theta =0^\circ$ calculated from the white line spectra.

Adsorbate	Edges	BR
Fe$_1$	Fe $L_{2,3}$	0.83±0.04
V$_1$	V $L_{2,3}$	0.66±0.03
Fe$_1$V$_1$	Fe $L_{2,3}$	0.79±0.03
	V $L_{2,3}$	0.58±0.03

Values for other incidence angles are omitted, as the branching ratio is found to be isotropic. For the alloy dimer, the branching ratio is shown for both elements separately.

From the white line spectra, the so called branching ratio (BR) was obtained as the area under the $L_3$ edge divided by the area under the integrated $L_3$ and $L_2$ edges. Table 1 summarizes the results for the normal incidence spectra shown in Figs. 1c–d and 4a–b. As the branching ratios obtained in this work are independent of the angle of incidence, we restrict ourselves to the values for $\theta =0^\circ$. We want to point out, that the determination of the branching ratio for all V species suffers from some uncertainty due to the overlap of the $L_3$ and $L_2$ edges and the somewhat arbitrary separation of the two. However, in the context of this work, it is possible to compare the obtained branching ratios as the edges were separated in the same way consistently.

Considering only spin-orbit splitting in the initial 2*p*-states of Fe and V, one would expect the absorption intensity from $j=3/2$ to make up 2/3 of the overall $2p_j\rightarrow 3d$ absorption intensity. This reflects the statistical degeneracy of the $2p_j$-levels. However, the presence of spin-orbit coupling in the initial 3*d*-states as well as electrostatic interactions of the 2*p* core-hole and the 3*d* final states tend to change the measured intensity ratio^15–17^. The adsorbed monomers’ branching ratios are, within the error bounds, identical to the ones obtained for free neutral atoms^11^ and cations^12^. They differ substantially from the branching ratios found for the respective bulk materials (BR(Fe)=0.70 and BR(V)=0.50)^16^. It has previously been established that increased screening of the 2*p* core, e.g. through hybridization with neighbouring atoms, lowers the branching ratio^18,19^. This is further evidence for a weak hybridization of the adatom and the substrate.

**Figure 2:**
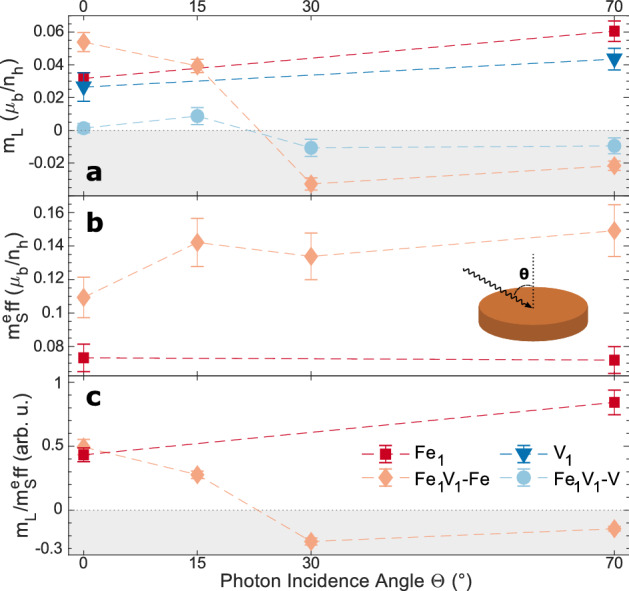
Orbital magnetic moments (**a**), effective spin magnetic moments (**b**) and ratio thereof (**c**) obtained via the sum rules^20,21^ for the investigated adsorbate systems as a function of photon incidence angle $\theta$. As the V $L_{2,3}$-edges are not separated well enough, only the orbital moments are calculated for those edges. The inset in (**b**) serves as a reminder of $\theta$ used in this work.

In the following, the magnetic properties of the V and Fe adatoms are discussed by means of the XMCD signals shown in Fig. 1(e–f). Using the well-established sum rules^20,21^, one can extract from the XMCD spectra the orbital magnetic moment m$_L$ and the effective spin magnetic moment m$_S^{eff}$=m$_S$+7m$_T$, where m$_T$ is the magnetic dipole moment, which accounts for non-spherical distributions of spin moments. The number of 3*d*-holes of the investigated atoms ($n_h$), which appears as a factor in the equations for the sum rules, is not attainable through XMCD measurements alone. As we have neither a theoretical nor experimental value for this factor, we report the sum rule results per $n_h$. The small energetic separation of the V *L*-edges would introduce a large error in the calculation of m$_S^{eff}$ and thus we only comment on the Fe spin moments. Although it is unlikely that the sample is in magnetic saturation, we can nevertheless refer to the values of the orbital and spin magnetic moments gained from the sum rules to compare measurements at different incidence angles, as they were all taken at the same magnetic field. Results of the sum rules applied to all spectra under investigation are plotted in Fig. 2.

For the V adatom, a weak but non-vanishing XMCD signal and hence a magnetic moment is found. This is in accordance with theoretical results^22^, where a magnetic moment of 3 $\mu _B$ was found for V/Cu(001), as well as experimental results for V embedded in Cu^23^. The $L_3$-XMCD-peak of the adsorbed V monomers XMCD is visibly stronger for $\theta =70^\circ$, indicating a preferred easy axis of magnetization which lies parallel to the Cu surface. This in-plane magnetization seen in the XMCD signal is confirmed by the anisotropy of the orbital moment (cf. Fig. 2**a**). For the Fe adatom, the height difference of the XMCD $L_3$-peaks is not so clear, and hence the orientation of the easy axis can not be determined as readily. However, the Fe orbital moment reveals a pronounced anisotropic behaviour favoring an in-plane orientation. This translates into an anisotropy in the $\mathrm {m}_L/\mathrm {m}_S^{eff}$ ratio (cf. Fig. 2**c**). With a value of 0.84$\pm 0.09$ for $\theta =70^\circ$,$\mathrm {m}_L/\mathrm {m}_S^{eff}$ is remarkably large and comparable to the one obtained for Fe on alkali metals, e.g. 0.95 for Fe/K^13^, where the large orbital magnetic moment was attributed to a weak hybridization with the substrate.

**Figure 3:**
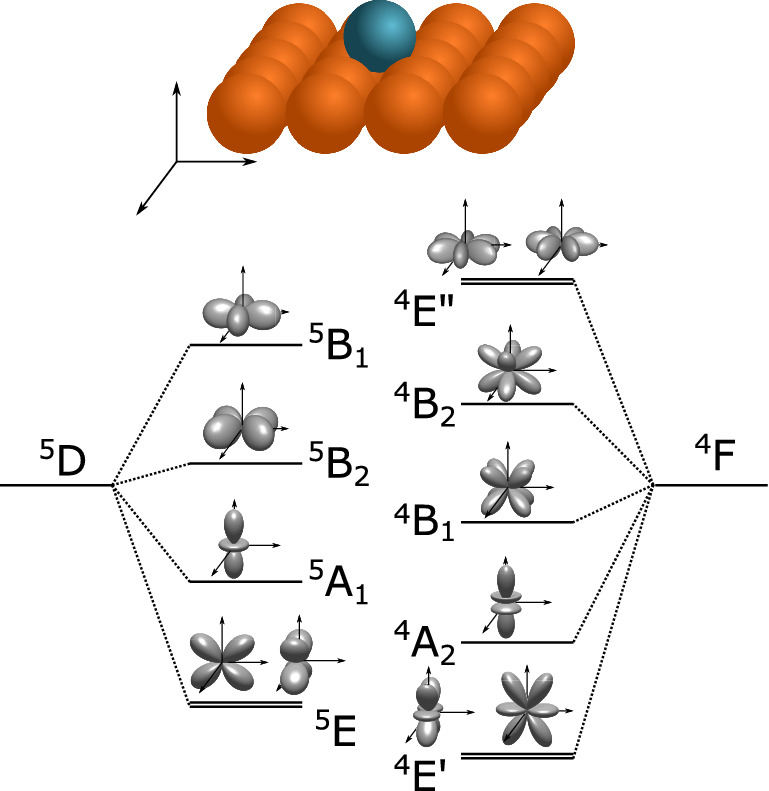
Sketch of the degeneracy lifting of the spherical symmetric $^5D$ and $^4F$ terms of the respective $d^6$ and $d^3$ configurations via the crystal field of a fourfold symmetric (001) surface and the form of the resulting irreducible representation. The new found representations are not ordered energetically.

Based on the measured in-plane anisotropy of both adatoms, we can gain further insight into the electronic ground state symmetry of the adsorbed monomers by using group theory. As both Fe and V are light transition metals with weak spin-orbit coupling^24^, they follow Russel-Saunders-coupling where the *l* and *s* quantum numbers of their 3*d* electrons will, in a first step, couple to form *L* and *S* values. This results in $^5D$ and $^4F$ terms for the respective $d^6$ and $d^3$ configurations of Fe and V. The crystal field of the fourfold symmetric Cu(001) surface will then split these terms into irreducible representations of the $C_{4v}$ point group. Geometric constraints only affect the spin through the spin-orbit coupling, thus one can focus primarily on the splitting of the orbital angular momentum. The $^5D$ term of $d^6$ will split into four different irreducible terms, each of which transforms like the one-electron orbital indicated in brackets: $^5\mathrm {A}_1$ ($d_{3z^2-r^2}$), $^5\mathrm {B}_1$ ($d_{x^2-y^2}$), $^5\mathrm {B}_2$ ($d_{xy}$) and the twofold degenerate $^5\mathrm {E}$ ($d_{xz}$ and $d_{yz}$). The $^4F$ term of $d^3$ will split into $^4\mathrm {A}_2$ ($f_{z^3}$), $^4\mathrm {B}_1$ ($f_{xyz}$), $^4\mathrm {B}_2$ ($f_{z(x^2-y^2)}$) and two E representations $^4\mathrm {E}'$ ($f_{yz^2}$ and $f_{xz^2}$) and $^4\mathrm {E}''$ ($f_{y(3x^2-y^2)}$ and $f_{x(x^2-3y^2)}$)^25^. This splitting is schematically shown in Fig. 3.

For 3*d* metals, the crystal field splitting is considerably larger than the spin-orbit splitting^4^. Thus, the terms arising from the symmetry lowering will not be mixed or rearranged by spin-orbit coupling and we can identify the $^4\mathrm {E}'$ and $^5\mathrm {E}$ terms as the respective ground states for the Fe and V adatom.

### Fe$_1$V$_1$/Cu(001)

**Figure 4:**
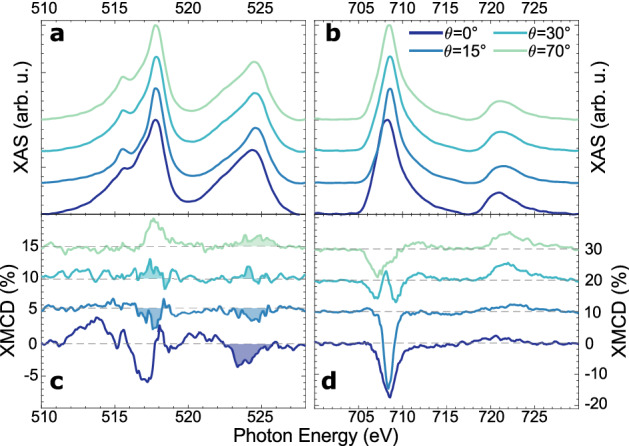
Background corrected white line spectra taken at $\theta$=0$^\circ$, $\theta$=15$^\circ$, $\theta$=30$^\circ$ and $\theta$=70$^\circ$ for the V $L_{2,3}$-edges (**a**) and the Fe $L_{2,3}$-edges (**b**) of Fe$_1$V$_1$/Cu(001). (**c**) and (**d**) are the XMCD spectra corresponding to (**a**) and (**b**). All spectra are normalized to the $L_3$ peak value of the corresponding white line spectrum and vertically offset for better visibility.

After having discussed the isolated Fe and V adatoms, we will bring both constituents together and study the Fe$_1$V$_1$ dimer. The white line spectra of both elements are shown in Fig. 4a–b for four different values of $\theta$, namely $0^\circ , 15^\circ , 30^\circ$ and $70^\circ$. This corresponds to an increased probing of in-plane molecular orbitals. Comparison of the normal incidence spectra with those of the adsorbed monomers shows a clear smearing of the multiplet features. We attribute this to an increased hybridzation and subsequent delocalization of the respective *d*-states in the dimer. This is consolidated by looking at Table 1, where a decrease in the branching ratio is observed for both species upon alloying. Due to the delocalization of the *d*-states, their overlap with the 2*p* core hole diminishes, which reduces both the branching ratio and the strength of the multiplets.

Furthermore, the peak positions of the white line spectra shift to lower energies for both elements. For Fe, the $L_3$ peak shifts from 709.0 eV to 708.4 eV, whereas the V $L_3$ changes from 518.0 eV to 517.8 eV upon alloying. This shift is significant, given the energy resolution of $\approx$30 meV of the P04 beamline at these photon energies^26^. Hirsch *et al.* have attributed such a lowering of the excitation energy to an increased dynamic screening of the 2*p* core hole by the 3*d* electrons in the final state^12^. The more efficient screening is a result of the stronger delocalization and subsequent increased mobility of the 3*d* electrons. This reduces the energy of the final state, leading to a lowering of the absorption energy. In addition to the overall shift, there is a noticeable energetic shift for Fe when going from $\theta =0^\circ$ to $\theta =15^\circ$. We attribute this to a stronger contribution of an energetically lower orbital at normal incidence, leading to a shifting center of mass of the whole peak^27,28^.

Focusing now on the XMCD spectra of both elements shown in Fig. 4c–d, it can be seen that the normal incidence XMCD spectrum of V shows a positive onset at the $L_3$ peak. In combination with the negative sign of the Fe $L_3$ XMCD in the same measurement geometry, this implies an antiferromagnetic coupling between the two constituents’ magnetic moments. This is in accordance with results for Fe/V/Fe trilayer systems^29^ and Fe$_{0.9}$V$_{0.1}$ alloys^30^, as well as electronic structure calculations of the free FeV dimer, where magnetic moments of $-2.8\,\mu _B$ and 1.8 $\mu _B$ were found for V and Fe respectively^31^. We want to note that the shape of the V XMCD is considerably different from the results of Wende *et al.*^29^ and Scherz *et al.*^30^. We explain this discrepancy by noting that the investigated system resembles an atomic species more than a bulk species and that subsequently the XMCD shows notably different features compared to the bulk^32,33^.

For $\theta =15^\circ$, the V XMCD signal decreases strongly in magnitude and for incidence angles larger than 15$^\circ$, there is a visible sign flip in the V XMCD (cf. shaded areas in Fig. 4c). This indicates a change from antiparallel to parallel orientation of the V magnetic moment relative to the magnetic field. The absence of such an inversion in the Fe XMCD shows that the coupling of the V and Fe magnetic moments is more complex than a collinear (antiferromagnetic or ferromagnetic) configuration. From Fig. 2a it can be seen that the orbital moments of Fe and V in the dimer have the same sign for all incidence angles and we can thus identify the spin-spin interaction of Fe and V as giving rise to the observed noncollinear alignment. $\theta =30^\circ$ also marks the appearance of a splitting of the $L_3$ peak in the Fe XMCD into two structures, which are well separated for $\theta =30^\circ$ and can still be distinguished for $\theta =70^\circ$. Similar to the energy shift of the XAS at $\theta =0^\circ$ a possible explanation for this is the increased contribution of dimer orbitals of differing magnetic signature when increasing $\theta$. Another possibility would be the existence of different adsorption sites of the Fe atoms of the dimer, which in turn couple differently to the V atoms.

To explain the noncollinear configuration, we make use of the two magnetic sublattices model used, for example, in^34^. Assuming an uniaxial anisotropy, the free energy of a dimer can be expressed by Eq. (1). Here, the first term is the Zeeman energy, the second term is the exchange energy between Fe and V and the third and fourth terms represent the anisotropy energies of the constituents up to second order.

1 $$ E =-B\varvec{\left( \right. }\mathrm {m_{Fe}}\cos (\alpha _{\rm Fe}-\theta )+\mathrm {m_V}\cos (\alpha _{\rm V}-\theta )\varvec{\left. \right) } -J_{\rm Fe-V} \mathrm {m_{Fe}} \mathrm {m_V}\cos (\alpha _{\rm Fe}-\alpha _{\rm V}) +K_{uni}^{\rm Fe}\sin ^2(\alpha _{\rm Fe})+K_{uni}^{\rm V}\sin ^2(\alpha _{\rm V}) $$

**Figure 5:**
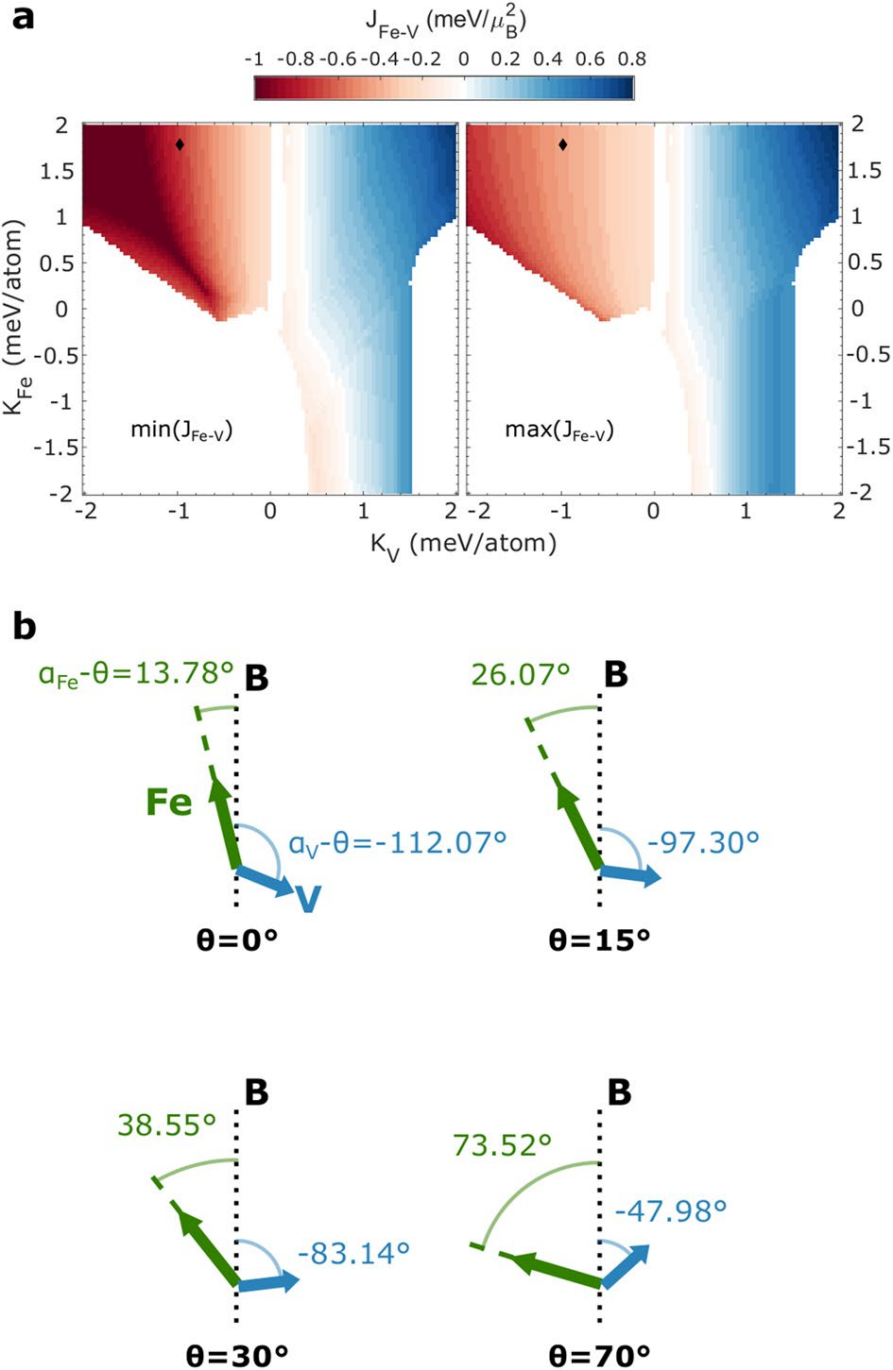
(**a**) Combinations of $J_{\text{Fe-V}}$, $K_{uni}^{\text{Fe}}$ and $K_{uni}^{\text{V}}$ which allow a spin-flop phase at a magnetic field strength of 7 T and magnetic moments of 2.27 $\mu _B$ and 0.99 $\mu _B$ for Fe and V respectively. For every pair of $K_{uni}^{\text{V}}$ and $K_{uni}^{\text{Fe}}$, all nonzero values of $J_{\text{Fe-V}}$ between the lower bound min($J_{\text{Fe-V}}$) (left) and the upper bound max($J_{\text{Fe-V}}$) (right) produce a noncollinear arrangement. The black diamond indicates the best fit to the observed XMCD height ratios. These are $J_{\text{Fe-V}}=$-0.575$\pm 0.005$ meV/$\mu _B^2$, $K_{uni}^{\text{Fe}}=$1.80$\pm 0.01$ meV/atom and $K_{uni}^{\text{V}}=$-0.97$\pm 0.01$ meV/atom. (**b**) Lowest energy orientations of the magnetic moments relative to the magnetic field, corresponding to the values indicated by the diamond in (**a**) for the four incidence angles used in this work.

$\alpha_{\rm Fe,V}$ denote the orientation angles of the magnetic moments $\mathrm {m_{Fe}}$ and $\mathrm {m_V}$ relative to the surface normal (cf. Fig. 5). $J_{\rm Fe-V}$ is the exchange constant between the two materials and $K_{uni}^{\rm Fe,V}$ are their respective anisotropy constants. A stable configuration is found by numerically minimizing Eq. (1) with respect to $\alpha _{\rm Fe}$ and $\alpha _{\rm V}$. Depending on the interplay between exchange coupling and anisotropy, there exists a so-called spin-flop phase in which the magnetic field, applied along the easy axis of the system, causes the magnetic moments to cant away from the collinear case.

In the absence of reported magnetic moments for the present system, $\mathrm {m_{Fe}}$ and $\mathrm {m_V}$ were assumed to be 2.27 $\mu _B$ and 0.99 $\mu _B$, based on the Fe$_{0.9}$V$_{0.1}$ alloy reported in^30^. Equation (1) was subsequently minimized for large ranges of $K_{uni}^{\rm Fe}$, $K_{uni}^{\rm V}$ and $J_{\rm Fe-V}$. The orientation of the magnetic moments is given by $\cos (\alpha _{\rm V,Fe}-\theta )$, meaning that a suitable set of constants should give $\cos (\alpha _{\rm V}-\theta )>0$ for $\theta \ge 30^\circ$.

An exemplary set of angles which were calculated to recreate the measurements are shown in Fig. 5(b). In this particular case, $J_{\text{Fe-V}}$ was -0.575$\pm 0.005$ meV/$\mu _B^2$, $K_{uni}^{\text{Fe}}$ was 1.80$\pm 0.01$ meV/atom and $K_{uni}^{\text{V}}$ was -0.97$\pm 0.01$ meV/atom. These values were chosen as they also yield similar magnitudes for the V XMCD in the case of $\theta =15^\circ$ and $\theta =30^\circ$. While $K_{uni}^{\text{Fe}}$ is significantly higher than the corresponding value of bcc Fe ($K_1\approx 4~\mu$eV/atom^35^), it is still reasonable considering the reduced symmetry of the Fe atom within the dimer. Also, $K_{uni}^{\text{Fe}}$ is remarkably similar to the value of *K* of 1.8 meV/atom found for Fe monomers on Cu(111)^8^. Isberg *et al.* found a combined $K_{uni}$ value of 0.3 meV for Fe/V(100) superlattices^36^. Although it is difficult to compare our results to this overall value, we want to note that the sum of $K_{uni}^{\text{Fe}}$ and $K_{uni}^{\text{V}}$ (namely 0.78 meV) turns out to be less than a factor of 3 off. Multiplying the best fit value of $J_{\text {Fe-V}}$ with the assumed magnetic moments of Fe and V, we arrive at an exchange energy of $-1.035$ meV, which is in reasonably good agreement with the antiferromagnetic exchange energy of -0.94 meV Poulopoulos *et al.* found for Fe$_n$V$_m$ superlattices on MgO(001)^37^.

## Conclusions

To summarize, we have studied the magnetic properties of Fe and V adatoms as well as the Fe$_1$V$_1$ dimer on a Cu(001) surface using X-ray spectroscopic methods. The Fe and V adatoms interact weakly with the Cu(001) surface, leading to the appearance of multiplet features in the $L_{2,3}$-edge NEXAFS spectra. From spectra at two different incidence angles, an in-plane easy axis has been seen for both adatoms. The respective orientations of the individual magnetic moments in the dimer have been determined by taking XMCD spectra at different photon incidence angles, i.e. angular resolved XMCD at the corresponding $L_{2,3}$-edges. These results give evidence for the existence of a spin-flop phase already in the magnetic moments in a Fe$_1$V$_1$ dimer on a Cu(001) surface. Employing a simple magnetic model, we were able to determine a set of constants that reproduce the measured behaviour. The magnitudes of these constants are consistent with the weak substrate-adsorbate interaction we found for the respective adatoms. The fundamental dimer system studied in this work already shows a complex noncollinear magnetic coupling and lends itself to the comparison to theoretical models, since many-body effects are less crucial in such a model system.

Going forward, it would be interesting to compare the present results with XMCD measurements on the free Fe$_1$V$_1$ dimer and to perform similar measurements on a wide range of dimer constituents and surfaces to establish systematic information on magnetic coupling at these scales.

## Methods

The preparation and deposition of monomers and dimers, as well as the X-ray absorption measurements were performed *in situ* and under UHV conditions at the P04 beamline^26^ at PETRA III in Hamburg. The low base pressure ($\le 5\times 10^{-10}$ mbar) during preparation and measurement is crucial in assuring that no oxidation of the adsorbates occurs, which would critically alter their electronic structure and magnetic properties. For the same reasons, and to ensure a reproducible substrate, the Cu surface was sputter cleaned and annealed extensively prior to each preparation.

Following^38^, adatoms and clusters were produced by sputtering a FeV target (50/50 at% with purity >99.9 %) with 30 keV Xe$^+$ ions. The cationic sputter fragments were extracted from the sputter region via a potential of 500 V. Mass selection of the fragments due to their mass-to-charge ratio was achieved in a dipole magnetic field perpendicular to the fragments trajectory. The resulting mass spectrum is shown in Fig. 1a. The mass-selected fragments are landed with a kinetic energy of $\sim$1 eV/atom and a soft landing scheme^39,40^ is employed to avoid fragmentation or implantation of the adsorbates into the substrate. Kr buffer layers were adsorbed onto the substrate at T$\approx$25 K before cluster deposition and desorbed afterward by flash heating to T$\approx$120 K. Figure 1b shows a schematic of the deposition process. The low substrate temperature of $\sim$25 K in combination with the low coverage of adsorbates on the surface (<0.03 ML), serve to prevent adsorbate diffusion and agglomeration.

The V $L_{2,3}$ and Fe $L_{2,3}$-edge spectra of the deposited species were recorded by scanning the photon energy in the ranges of 500–528 eV and 700–730 eV, respectively. For the present case, the energy resolution of the beamline was $\sim$30 meV^26^. The sample drain current, representing the total electron yield (TEY), was used as a measure of the X-ray absorption. To minimize the influence of possible fluctuations of the photon flux on the absorption, measurements were normalized to the TEY signal of an Au grid placed half a meter before the sample.

The XMCD signal was obtained as the difference of absorption spectra for parallel ($\mu _+$) and antiparallel ($\mu _-$) alignment of the photon wavevector $\mathbf {k}$ and the magnetic field $\mathbf {B}$. In contrast to our earlier work on mass-selected adsorbates^41–44^, alignment of the magnetic moments was achieved via a superconducting split pair solenoid magnet with maximum magnetic field of ±7 T, purchased from *Cryogenic Limited*^45^. The polarization of the photon beam was kept fixed and collinear to the magnetic field, which was switched between the two extrema. Angular dependence of the adsorbate magnetic moments was investigated by rotating the sample, while keeping the respective directions of the photon wavevector $\mathbf {k}$ and the magnetic field $\mathbf {B}$ fixed, and hence changing the photon angle of incidence relative to the surface normal ($\theta$ in Fig. 1b).

Given the dilute nature of the adsorbates, the absorption spectrum is dominated by the Cu substrate. As the investigated energy range lies far away from an X-ray absorption edge of the substrate, its contribution is treated as a linear background and subtracted from the spectra. Furthermore, a double step function was subtracted from all the spectra accounting for the absorption signal from Fe and V electrons being excited into *sp*-like states.
